# Vagal α7nAChR signaling regulates α7nAChR^+^Sca1^+^ cells during lung injury repair

**DOI:** 10.1186/s13287-020-01892-4

**Published:** 2020-08-31

**Authors:** Xiaoyan Chen, Jie Chen, Yuanlin Song, Xiao Su

**Affiliations:** 1 Department of Pulmonary and Critical Care Medicine, Zhongshan Hospital, Fudan University and Shanghai Respiratory Research Institute, 180 Fenglin Road, Shanghai, 200032 People’s Republic of China; 2 Unit of Respiratory Infection and Immunity, Institut Pasteur of Shanghai, Chinese Academy of Sciences, 320 Yueyang Road, Shanghai, 200031 People’s Republic of China; 3 Department of Pulmonary and Critical Care Medicine, Zhongshan Hospital, Qingpu Branch, Shanghai, People’s Republic of China; 4 National Clinical Research Center for Aging and Medicine, Huashan Hospital, Fudan University, Shanghai, People’s Republic of China

**Keywords:** Vagal circuits, α7nAChR, Sca1^+^ cells, Acute lung injury, Repair

## Abstract

**Background:**

The distal airways of the lung and bone marrow are innervated by the vagus nerve. Vagal α7nAChR signaling plays a key role in regulating lung infection and inflammation; however, whether this pathway regulates α7nAChR^+^Sca1^+^ cells during lung injury repair remains unknown. We hypothesized that vagal α7nAChR signaling controls α7nAChR^+^Sca1^+^ cells, which contribute to the resolution of lung injury.

**Methods:**

Pneumonia was induced by intratracheal challenge with *E. coli*. The bone marrow mononuclear cells (BM-MNCs) were isolated from the bone marrow of pneumonia mice for immunofluorescence. The bone marrow, blood, BAL, and lung cells were isolated for flow cytometric analysis by labeling with anti-Sca1, VE-cadherin, p-Akt1, or Flk1 antibodies. Immunofluorescence was also used to examine the coexpression of α7nAChR, VE-cadherin, and p-Akt1. Sham, vagotomized, α7nAChR knockout, and Akt1 knockout mice were infected with *E. coli* to study the regulatory role of vagal α7nAChR signaling and Akt1 in Sca1^+^ cells.

**Results:**

During pneumonia, BM-MNCs were enriched with α7nAChR^+^Sca1^+^ cells, and this cell population proliferated. Transplantation of pneumonia BM-MNCs could mitigate lung injury and increase engraftment in recipient pneumonia lungs. Activation of α7nAChR by its agonist could boost α7nAChR^+^Sca1^+^ cells in the bone marrow, peripheral blood, and bronchoalveolar lavage (BAL) in pneumonia. Immunofluorescence revealed that α7nAChR, VE-cadherin, and p-Akt1 were coexpressed in the bone marrow cells. Vagotomy could reduce α7nAChR^+^VE-cadherin^+^ and VE-cadherin^+^p-Akt1^+^ cells in the bone marrow in pneumonia. Knockout of α7nAChR reduced VE-cadherin^+^ cells and p-Akt1^+^ cells in the bone marrow. Deletion of Akt1 reduced Sca1^+^ cells in the bone marrow and BAL. More importantly, 91.3 ± 4.9% bone marrow and 77.8 ± 4.9% lung α7nAChR^+^Sca1^+^VE-cadherin^+^ cells expressed Flk1, which is a key marker of endothelial progenitor cells (EPCs). Vagotomy reduced α7nAChR^+^Sca1^+^VE-cadherin^+^p-Akt1^+^ cells in the bone marrow and lung from pneumonia mice. Treatment with cultured EPCs reduced ELW compared to PBS treatment in *E. coli* pneumonia mice at 48 h. The ELW was further reduced by treatment with EPCs combining with α7nAChR agonist-PHA568487 compared to EPC treatments only.

**Conclusions:**

Vagal α7nAChR signaling regulates α7nAChR^+^Sca1^+^VE-cadherin^+^ EPCs via phosphorylation of Akt1 during lung injury repair in pneumonia.

## Introduction

Severe pneumonia is a common cause of acute respiratory failure and acute lung injury (ALI)/acute respiratory distress syndrome (ARDS). Despite the introduction of effective antibiotics and intensive supportive care in the twentieth century, death rates from community-acquired pneumonia among patients in the intensive care unit remain as high as 35% [1]. So far, no specific therapy was found to treat ALI/ARDS. Novel therapies are needed to address this problem.

The vagal nerve is the dominant nerve of the distal airway of the lung, including the alveoli [2, 3]. Vagal sensory nerve endings, brain integration centers, acetylcholine, and α7 nicotinic acetylcholine receptor (nAChR)-expressing cells form a pulmonary parasympathetic inflammatory reflex to regulate lung infection and inflammation [4, 5]. This machinery also synergizes with the spleen (as a functional hub of the cholinergic anti-inflammatory pathway) to fine-tune lung infection and immunity [6]. Clinical studies have demonstrated that vagus nerve stimulation targeting the inflammatory reflex modulates the TNF production and reduces inflammation in rheumatoid arthritis [7]. Vagotomy after traumatic injury is associated with an increase in ulcer disease, septicemia, and mortality [8]. Recently, we reported that vagal α7nAChR signaling could promote lung stem cell regeneration via fibroblast growth factor 10 during lung injury repair [9]. Whether the vagus nerve regulates the bone marrow-derived progenitor (Sca1^+^) cells during pneumonia is unclear.

The BM is richly innervated with both myelinated and nonmyelinated nerve fibers. In silver-stained marrow preparations and electron microscopic examinations, the majority of fibers are associated with the nutrient vessels and the central sinus of the marrow, with some fibers penetrating into the parenchyma [10–12]. Hematopoietic stem cells and mesenchymal stem cells (MSCs) express α7nAChR [13–15]. Endothelial progenitor cells (EPCs) also express α7nAChR, and stimulating α7nAChR by nicotine can improve the migration of EPCs [16] and promote repair [17, 18]. However, whether vagal circuits regulate α7nAChR^+^Sca1^+^ cells in the bone marrow and lung and the underlying mechanisms during pneumonia are unknown.

The PI3 (phosphatidylinositol-3) kinase/Akt (protein kinase B) pathway is involved in molecular signaling that regulates retrograde axonal transport of neurotrophins in the vagus nerve [19]. Nicotine-induced neuroprotection is mediated by the PI3K/Akt pathway [20]. Nicotine can also increase the numbers and activity of endothelial progenitor cells by augmenting telomerase activity via the PI3K/Akt pathway [21]. PI3K and Fyn are physically associated with α7nAChR [20, 22, 23]. Therefore, Akt1 might be involved in the regulation of α7nAChR^+^Sca1^+^ cells in the bone marrow and lung during lung injury repair.

Adult progenitor cells played a potential therapeutic role in lung repair. Bone marrow-derived progenitor cells (BMPCs) are important and required for lung repair after LPS-induced lung injury [24]. BMPCs could sense LPS and then generate S1P to stabilize endothelial junction barrier [25] or be induced to differentiate into MSCs to improve survival [26] or be induced to differentiate to EPCs to attenuate lung injury in acute lung injury [27]. EPCs also have been reported to reduce lung injury in septic mice and rats [28, 29]. EPCs, characterized by the cell surface markers CD45(^dim/−^), CD133^+^, VE-cadherin (CD144)^+^, and FLK1 (VEGF receptor 2)^+^, can be quantified by flow cytometry [30]. Embryonic stem cells express VE-cadherin (CD144) during endothelial differentiation [31]. Neuropeptide (CGRP) can significantly increase the fraction of CD31^+^CD144^+^ EPCs, and the capillary density in the bone defect at the end of the distraction phase [32]. Therefore, we proposed that α7nAChR^+^Sca1^+^ cells expressing VE-cadherin might be EPCs. Whether vagal α7nAChR signaling can modulate these cells between the bone marrow and lung during pneumonia needs to be determined. The results of this study will deepen our understanding of neural regulatory mechanisms that endogenous bone marrow EPCs repair ALI/ARDS.

Hence, the objectives of this study were (i) to study changes in α7nAChR^+^Sca1^+^ cells in the bone marrow and lung during pneumonia, (ii) to determine whether transplantation of α7nAChR^+^Sca1^+^ cell-enriched BM-MNCs affects lung injury, (iii) to examine whether vagotomy has an impact on α7nAChR^+^VE-cadherin^+^ and VE-cadherin^+^p-Akt1^+^ cells, and (iv) to elucidate whether vagotomy affects α7nAChR^+^Sca1^+^VE-cadherin^+^p-Akt1^+^ cells in pneumonia mice. Our findings indicate that vagal circuits regulate α7nAChR^+^Sca1^+^VE-cadherin^+^ EPCs via phosphorylation of Akt1. For the first time, we identified the bone marrow α7nAChR-expressing EPCs, which could be regulated by vagal α7nAChR-p-Akt1 signaling for lung injury repair.

## Materials and methods

### Reagents

DMAB-anabasine dihydrochloride and PHA568487 were obtained from Tocris Biosciences (Minneapolis, MN, USA); (−)-nicotine hemisulfate salt, and *Escherichia coli* 0111:B4 lipopolysaccharide was purchased from Sigma (St. Louis, MO). Anti-mouse CD16/CD32 monoclonal antibody (IM7) was purchased from eBioscience (San Diego, CA, USA). PE rat anti-mouse Ly-6A/E (clone D7) was purchased from BD Biosciences (San Jose, CA, USA). Phospho-Akt1 (Ser473) (D7F10) XP® rabbit mAb (Akt1 Specific) and PathScan® Phospho-Akt1 (Ser473) Sandwich ELISA Kit were obtained from Cell Signaling (Danvers, MA, USA). CF633 α-Bungarotoxin was purchased from Biotium (Fremont, CA, USA). α7nAChR antibody (H-302) was purchased from Santa Cruz Biotechnology (Santa Cruz, CA, USA). BV421 rat anti-mouse CD144 was purchased from BD Horizon. Alexa Fluor 488 anti-mouse CD309 (VEGFR2, Flk-1) antibody was purchased from BioLegend. The *E. coli* K1 (serotype) strain, isolated from patients with biliary infection, was kindly provided by Dr. Thomas Martin (University of Washington, USA) [33].

### Animals

α7nAChR knockout (α7nAChR^−/−^, background, C57BL/6J, B6.129S7-*Chrna7*tm1Bay/J, stock no. 003232) and *Akt1*^*−/−*^mice (backcrossed to a C57BL/6 background for 6 generations) were purchased from Jackson Laboratory (Bar Harbor, ME, USA) [2]. Littermate wild-type mice (C57BL/6J background, 6–8 weeks old) were used as controls. We only used male mice in the study considering that estrogen in females may influence the effect of the bone marrow-derived progenitor cells [34]. The mice were housed with free access to food and water in 12-h dark/light cycle. Anesthetization was performed by intraperitoneal injection of pentobarbital sodium (50 mg/kg). The protocols were approved by the Committees on Animal Research of the Institut Pasteur of Shanghai, Chinese Academy of Sciences, China.

### *E. coli* pneumonia model

The methods used to passage, store, amplify, and quantify the bacteria have been described [6]. Acute lung injury was induced by instilling *E. coli* into the lungs of mice [6]. *E. coli*, 2.5 × 10^6^ CFU, was used for a longer period of the experiment, for example, 24 h, to ensure that there was no death in either the control or treated groups. For experiments with *Akt1*^*−/−*^ and wild-type mice (C57BL/6J), 10^6^ CFU *E. coli* were intratracheally challenged.

### Unilateral vagotomy

Cervical vagotomy was performed as described previously [6]. Briefly, a longitudinal midline incision was made in the ventral region of the neck before blunt dissection. The overlying muscles and fascia were separated until the right vagus was visible. For the vagotomy (Vx) group, the vagus was carefully stripped away from the carotid artery and lightly cutoff. For the sham group, the vagus was left intact. The wound was closed and sutured. The protocols were approved by the Committees on Animal Research of the Institut Pasteur of Shanghai, Chinese Academy of Sciences.

### Animal treatments

In an LPS-induced ALI or *E. coli* pneumonia mouse model, the α7nAChR agonists, PHA568487 (0.4 mg/kg, ip, q6h), nicotine (0.4 mg/kg, ip, q6h), or DMAB (0.4 mg/kg, ip, q8h) were administered as described previously [35]. The first dosage was given 15 min before LPS or *E. coli* challenge.

### Measurement of extravascular lung water (ELW)

The gravimetric method was used to determine ELW as previously described [2]. Homogenate and supernatant of the lung and blood were weighed and then desiccated in an oven (60 °C for 24 h). ELW was calculated by the standard formula [2]. The controls were normal mice of the same age as the experimental group.

### ELISA measurements of interleukin 10 (IL-10) and stem cell factor (SCF) in lung homogenates

IL-10 and SCF concentrations were measured in supernatants of lung homogenates with ELISA kits (R&D Systems).

### RNA isolation and RT-PCR

RNA was isolated from the bone marrow using the Qiagen RNAeasy kit (Qiagen Inc., CA). RT-PCR was performed using the SuperScript III One-Step RT-PCR System with the Platinum Taq DNA Polymerase protocol from Invitrogen according to the manufacturer’s instructions in a reaction volume of 25 μl. For α7nAChR DNA amplification, an initial reverse transcription step (52 °C for 30 min) was followed by a denaturing step (94 °C for 2 min) and then 40 cycles of denaturing (94 °C for 20 s), annealing (60 °C for 30 s), and extending (68 °C for 30 s), followed by 5 min at 72 °C for a final elongation. To normalize the loading of the PCR products, the *GAPDH* gene was amplified as an internal control (RT 58 °C for 30 min, denaturation at 94 °C for 2 min, 18 cycles of amplification at 94 °C for 20 s, 64 °C for 30 s, and 68 °C for 30 s, and elongation at 68 °C for 5 min). Primers were *Chrna 7* Forward: ACATTGACGTTCGCTGGTTC; Reverse: TACGGCGCATGGTTACTGT, 235 bp; *Gapdh* Forward: AATGGATTTGGACGCATTGGT; Reverse: TTTGCACTGGTACGTGTTGAT, 213 bp.

### Isolation of mononuclear cells from the bone marrow and peripheral blood

The bone marrow from the femurs and tibias was flushed with 2% FCS DMEM (Gibco) using a 25-gauge needle. The cells were dispersed and filtered over a 70-μm nylon cell strainer (BD Biosciences-Discovery Labware). The cell pellets were resuspended in 2% FCS DMEM, and then, 4 ml of solution was carefully layered on 3 ml of Ficoll (Ficoll-Paque™ PLUS, GE Healthcare). For isolation of peripheral blood mononuclear cells, EDTA anticoagulated blood was diluted 1:2 with Ca^2+^ Mg^2+^-free HBSS (Gibco) and then carefully layered on Ficoll. Samples were centrifuged for 25 min at 700×*g* and 22 °C without applying a brake. The PBMC interface was carefully removed by pipetting and washed twice with HBSS by stepwise centrifugation for 15 min at 300×*g* and for 10 min at 90×*g* for platelet removal.

### Culture of endothelial progenitor cells

Mouse EPCs were cultured the methods as previously reported [25, 36]. Briefly, the femur and tibia were collected to isolate the bone marrow cells. Mononuclear cells were isolated from the bone marrow cells by density gradient (Ficoll-Paque; Amersham) centrifugation. The cells were resuspended in EBM-2MV endothelial culture basal media using the supplement kit (Lonza) made of 10% FBS, 50 U/ml penicillin and streptomycin, 2 mmol/l l-glutamine (Invitrogen), and additional VEGF (5 ng/ml). The cells were then plated onto fibronectin-collagen-gelatin (1:1:1)-coated tissue culture flasks. The cells were incubated for 48 h at 37 °C with 5% CO_2_, at which time the nonadherent cells, representing 90–95% of the initial culture, were washed away. The adherent cells were then cultured for 14 days. The phenotype of the confluent cell population was assessed by determining by EPC markers by Immunofluorescence and flow cytometry.

### Bronchoalveolar lavage

The detailed procedures were described previously [2, 35]. BAL cells were also used for flow cytometry after lysis of erythrocytes.

### Isolation of lung cells

Lung single-cell suspensions were prepared each time using 3–5 mice as described [37] with modification. In brief, 1 ml dispase (2 U/ml) was injected through the trachea. Subsequently, the trachea was removed, and the lungs were minced and incubated in a 37 °C shaking incubator for 45 min in 2 ml of 2 μg/ml collagenase/dispase containing 0.001% DNase. The lung suspensions were filtered through 40 μm cell strainers, centrifuged, and depleted of red blood cells using RBC lysis buffer. Subsequently, lung Sca1^+^ cells were analyzed by flow cytometry.

### PKH67 labeling of stem cells and their tracking in lungs

Isolated BM-MNCs were labeled with PKH67 (Sigma-Aldrich) according to the manufacturer’s protocol. The lungs were collected at the endpoints of experiments, embedded in OCT (Sakura FineTek, Torrance, CA, USA), and frozen in liquid nitrogen. Frozen sections (5 μm) were prepared in a cryostat (MICROM HM 505E), analyzed for fluorescence using a FITC filter on a fluorescence microscope (Axioscop 2 fitted with Axiocam HRC, ZEISS, CH), and digitized using AxioVision software v 4.0.

### Immunofluorescence

Using cytospin, the bone marrow mononuclear cells were prepared on slides and fixed in 4% paraformaldehyde. The smear was permeabilized in 0.2% Triton and incubated with the following monoclonal antibodies: 1:100 rabbit anti-mouse α7nAChR and 1:100 rat anti-mouse Sca1 (BD Pharmingen). Then, anti-rabbit or rat Fluor 594 or FITC-labeled secondary antibody (Molecular Probes) was added to the smear and incubated for 1 h. After washing with hypertonic PBS (2.7% NaCl), the slides were mounted with ProLong Gold antifade reagent (Molecular Probes). For the bone marrow immunofluorescence, we carefully harvested the bone marrow, embedded it in OCT (Sakura FineTek, Torrance, CA, USA), and froze it in liquid nitrogen. Frozen sections (5 μm) were prepared in a cryostat (MICROM HM 505E). The sections were incubated with anti-α7nAChR, VE-cadherin, and p-Akt1 fluorescent antibodies and corresponding secondary fluorescent antibodies. All staining procedures were performed with appropriate isotype controls.

### Flow cytometry

After incubating for 15 min with an anti-mouse CD16/32 antibody, BM, blood, BAL, and lung cells were labeled with primary or isotype antibodies. Isotype antibodies and unstained controls were used to demonstrate the specificity of staining and to establish the criteria for target populations (for simplicity, the data regarding these controls are not shown). Debris and aggregates were excluded, and live cells were analyzed by LSRFortessa (BD Biosciences, San Jose, CA, USA). Data were analyzed by FlowJo vX.0.7 software (Tree Star Inc., Ashland, OR, USA).

### Statistical analysis

Statistical analyses were performed using SPSS software (SPSS Inc., Chicago, IL). An unpaired *t* test was used unless there were multiple comparisons, in which case we used ANOVA with post hoc Bonferroni test (significance level set at *p* < 0.05).

## Results

### α7nAChR^+^Sca1^+^ cells are increased in the bone marrow during lung injury repair

**Fig. 1:**
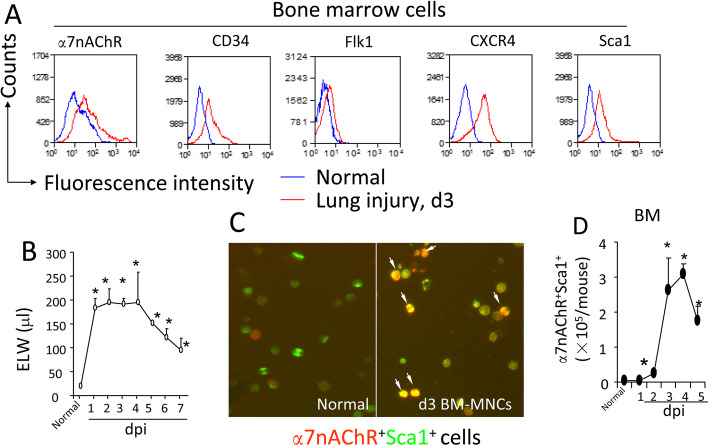
Flow cytometric and immunofluorescent analysis of the bone marrow α7nAChR-expressing progenitor cells. **a** Flow cytometric analysis of the bone marrow α7nAChR-, CD34-, Flk1-, CXCR4-, and Sca1-expressing cells. The bone marrow mononuclear cells were isolated from normal mice and pneumonia mice at 3 days after intratracheal *E. coli* challenge and stained with anti-α7nAChR, CD34, Flk1, CXCR4, and Sca1 fluorescent antibodies. Cells were analyzed in the lymphocyte gate. Representative results from individual mice are shown. **b** Dynamic changes in extravascular lung water (ELW) in *E. coli* pneumonia. Mice were intratracheally challenged with *E. coli* (2.5 × 10^6^ CFU) and sacrificed for 7 consecutive days. The lungs were collected to measure ELW, an index of pulmonary inflammation and edema. *N* = 3–5 in each group. Two-way ANOVA was used. ^*^P < 0.05 versus normal control. **c** Immunofluorescent detection of BM α7nAChR^+^Sca1^+^ cells. BM-MNCs isolated from normal and pneumonia mice at 3 dpi. The cells were subjected to immunofluorescent staining. **d** Flow cytometry analysis of dynamic changes in BM α7nAChR^+^Sca1^+^ cells. Mice were intratracheally challenged with *E. coli* (2.5 × 10^6^ CFU) and sacrificed for 7 consecutive days. BM cells were isolated, stained with anti-α7nAChR and Sca1 fluorescent antibodies, and subjected to flow cytometry. Normal BM cells were used as controls. *N* = 3–5 in each group. Two-way ANOVA was used. ^*^P < 0.05 versus normal control. Data are shown as the mean ± SD

We first compared α7nAChR^+^, CD34^+^, Flk1^+^, CXCR4^+^, and Sca-1^+^ cell populations in the bone marrow between normal and d3 *E. coli* pneumonia mice. These cell populations were increased in the d3 *E. coli* pneumonia group compared to the normal group (Fig. 1a). To study the dynamics of extravascular lung water (ELW), we intratracheally challenged the mice with 2.5 × 10^6^ CFU live *E. coli* and sacrificed them consecutively for 7 days. ELW was increased 12-fold, plateaued from d2–4, and decreased from d5 (Fig. 1b). To examine changes in the bone marrow α7nAChR^+^Sca1^+^ cells, we isolated the bone marrow mononuclear cells (BM-MNCs) from normal and d3 *E. coli* pneumonia mice and performed immunofluorescence. The bone marrow α7nAChR^+^Sca1^+^ cells were increased in d3 *E. coli* pneumonia mice (Fig. 1c). To dynamically observe changes in the bone marrow α7nAChR^+^Sca1^+^ cells in response to lung injury, mice were intratracheally challenged with 2.5 × 10^6^ CFU live *E. coli* and sacrificed in the 5 subsequent days. Normal mice were used as controls. By flow cytometry analysis, we found that the bone marrow α7nAChR^+^Sca1^+^ cells were increased at d3–5 in the *E. coli* pneumonia group (Fig. 3d). These findings suggest that α7nAChR^+^Sca1^+^ cells might be involved in the repair of lung injury.

### Anti-inflammatory and engraftment properties of BM-MNCs from pneumonia mice

**Fig. 2:**
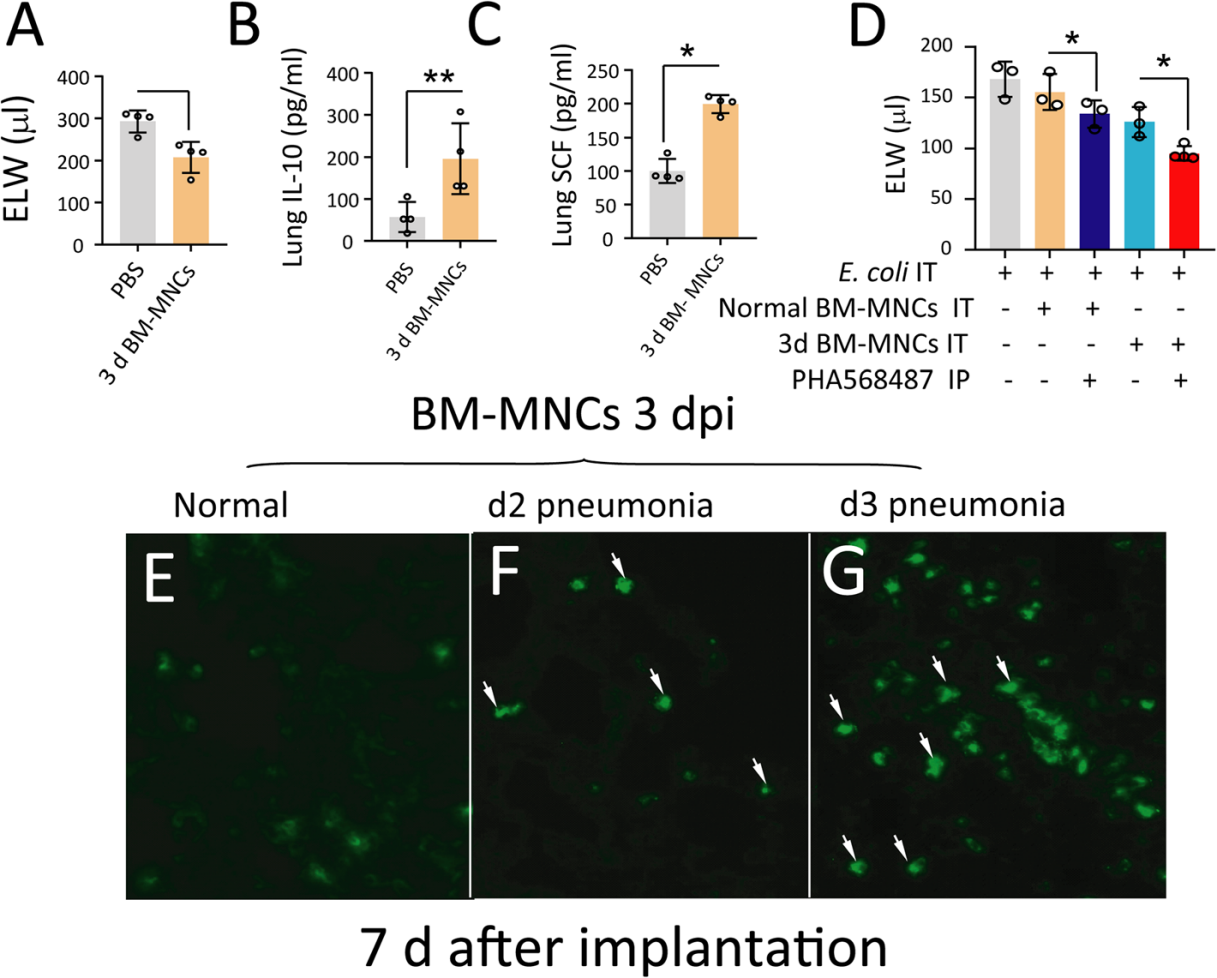
Effects of the combination of BM-MNCs and α7nAChR agonist (nicotine) on *E. coli* pneumonia. **a**–**c** Effects of intrapulmonary delivery of 3-day BM-MNCs on ELW, lung IL-10, and SCF in recipient mice with acute lung injury induced by *E. coli*. Student’s *t* test was used. ^*^P < 0.05, ^**^P < 0.01 versus the PBS group. *N* = 4 mice in each group. Data are shown as the mean ± SD. **d** Effects of the activation of α7nAChR by nicotine in BM-MNCs from d3 pneumonia mice on recipient pneumonia mice. One-way ANOVA was used. ^*^P < 0.05, *N* = 3–4 mice in each group. **e**–**g** Engraftment of BM-MNCs obtained from d3 pneumonia mice in the recipient lungs with *E. coli* pneumonia. The recipient mice were sacrificed 7 days after BM-MNC transplantation. **e** Normal recipient mice; **f** d2 pneumonia recipient mice; **g** d3 pneumonia recipient mice. *N* = 3 mice in each group

To test whether BM-MNCs modulate lung inflammation, d3 BM-MNCs (10^6^ cells in 25 μl PBS, isolated from the bone marrow of donor mice with *E. coli* pneumonia at 3 days) were delivered into the lungs of recipient mice 4 h after *E. coli* challenge (2.5 × 10^6^ CFU). The control mouse group received PBS. All recipients were sacrificed 2 days later. ELW was reduced in the lungs of mice receiving BM-MNCs from mice with pneumonia (Fig. 2a). Lung IL-10 (an anti-inflammatory cytokine) and SCF (a growth factor required for survival, proliferation, and differentiation of HSCs) levels in the recipient lung homogenates were significantly increased in the pneumonia mice receiving d3 BM-MNCs (Fig. 2a–c). These findings suggest that activation of α7nAChR might promote repair of lung injury via the bone marrow α7nAChR^+^Sca1^+^ cells. Since d3 BM-MNCs were enriched with α7nAChR^+^Sca1^+^ cells, we isolated BM-MNCs (10^6^ cells) from d3 pneumonia and normal mice and transplanted these cells (10^6^ cells) into the lungs of recipient mice 4 h after *E. coli* challenge (2.5 × 10^6^ CFU) in combination with or without PHA568487 (α7nAChR agonist). We observed a decrease in ELW in d3 BM-MNC recipient mice compared to normal BM-MNC recipient mice. ELW was further reduced in PHA568487-treated d3 BM-MNC recipient mice compared to untreated d3 BM-MNC recipient mice (Fig. 2d). To determine the engraftment properties of the d3 BM-MNCs, these cells were isolated from donor pneumonia mice, labeled with green fluorescent dye (PKH67-GL, Sigma), and then intratracheally instilled into recipient lungs of normal (Fig. 2e), d2 (Fig. 2f), or d3 pneumonia mice (Fig. 2g). After 7 days, the recipient mice were sacrificed, and lung immunofluorescence was performed to examine green cells implanted in the lung tissue. We found that engraftment in the 3 days recipient lungs was significantly higher than that in the other two groups (Fig. 2e–g). The findings indicate that BM-MNCs containing α7nAChR^+^Sca1^+^ cells possess engrafting properties.

### Activation of α7nAChR increases α7nAChR^+^Sca1^+^ cells from the bone marrow, blood, and BAL in *E. coli* pneumonia

**Fig. 3:**
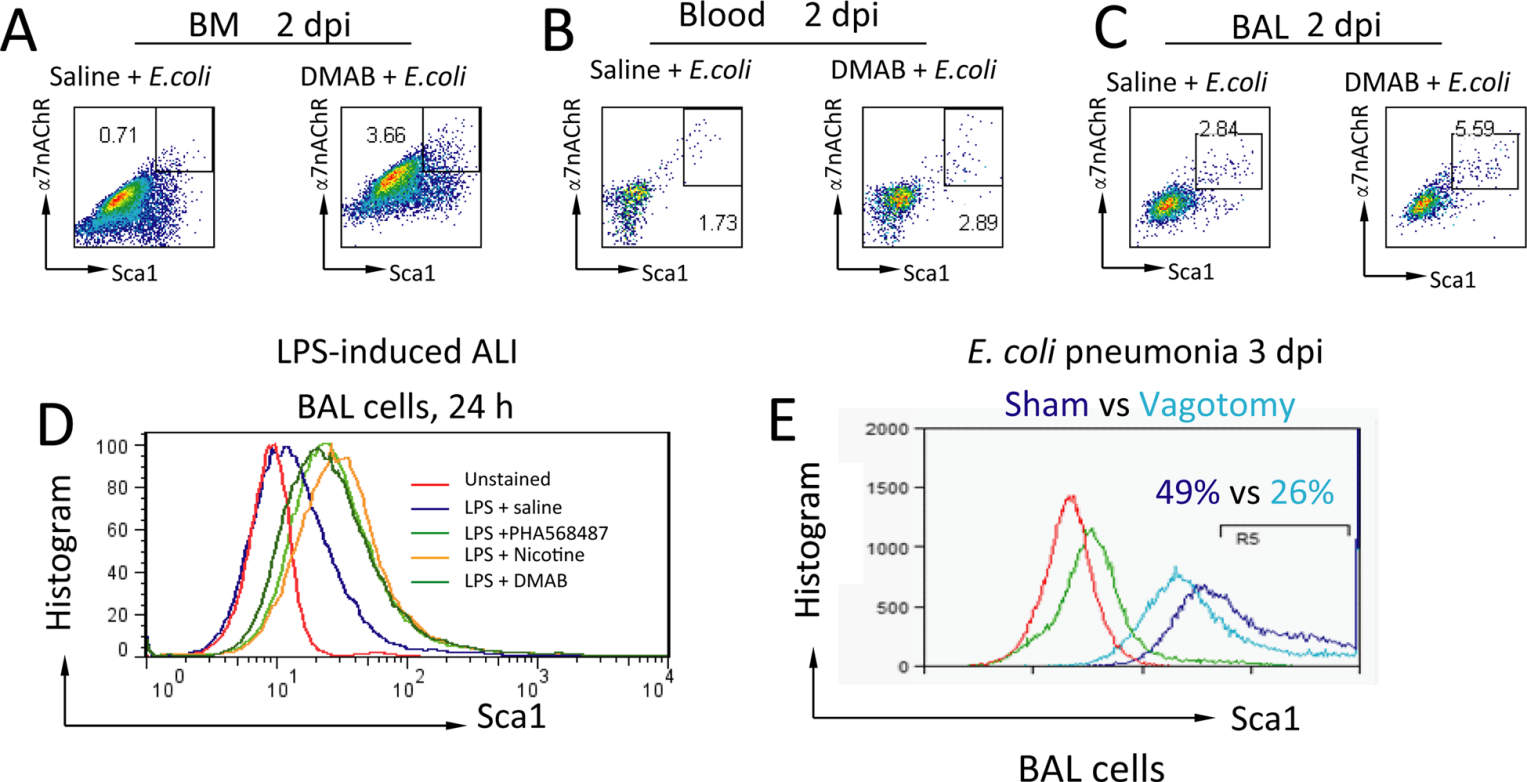
Effects of the activation of α7nAChR by DMAB or vagotomy on α7nAChR^+^Sca1^+^ and Sca1^+^ cells in the bone marrow, peripheral blood, and BAL in pneumonia. **a**–**c** Effects of the α7nAChR agonist DMAB on α7nAChR^+^Sca1^+^ cells in the bone marrow, blood, and BAL in *E. coli*-induced acute lung injury at 2 dpi. **d** Effects of the α7nAChR agonists PHA568487, DMAB, and nicotine on Sca1^+^ cells in BAL with LPS-induced acute lung injury at 24 h. **e** Effects of vagotomy on Sca1^+^ cells in BAL in pneumonia at 3 dpi. Red line: unstained cells; green line: normal cells. Data are representative of three independent experiments

We next tested whether activation of α7nAChR could boost α7nAChR^+^Sca1^+^ cells from the bone marrow, blood, and BAL in *E. coli* pneumonia mice treated with the α7nAChR agonist DMAB (0.4 mg/kg, ip, q8h) or saline for 2 dpi. The bone marrow, blood MNCs, and BAL cells were isolated and labeled with corresponding fluorescent antibodies for flow cytometry. We found that α7nAChR^+^Sca1^+^ cells were increased in the bone marrow, blood, and BAL cells from DMAB-treated *E. coli* pneumonia mice (Fig. 3a–c). In an LPS-induced acute lung injury mouse model (LPS 5 mg/kg, IT), mice were treated with saline, PHA568487 (0.4 mg/kg, ip, q6h), nicotine (0.4 mg/kg, ip, q6h), or DMAB (0.4 mg/kg, ip, q8h) for 24 h. BAL cells were isolated, and Sca1^+^ cells were analyzed by flow cytometry. Sca1^+^ cells were increased in all α7nAChR agonist-treated groups compared to the LPS + saline-treated group (Fig. 3d). In *E. coli* pneumonia, vagotomy also reduced BAL Sca1^+^ cells compared to sham mice at 3 dpi (Fig. 3e). These findings support the hypothesis that activation of α7nAChR can boost α7nAChR^+^Sca1^+^ cells during lung injury.

### Activation of α7nAChR regulates α7nAChR^+^VE-cadherin^+^ bone marrow cells in *E. coli* pneumonia

**Fig. 4:**
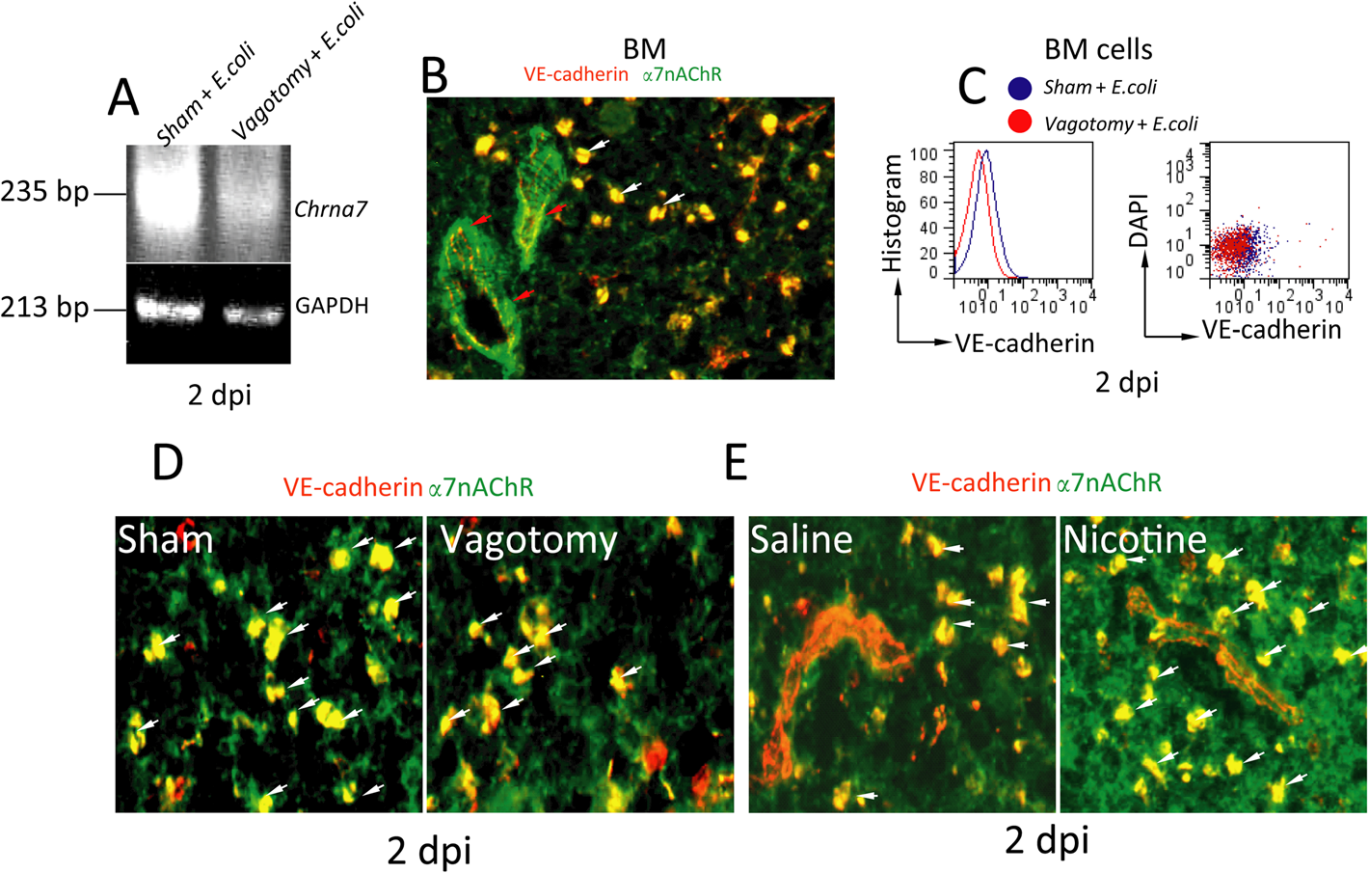
Effects of vagotomy or α7nAChR knockout on α7nAChR^+^VE-cadherin^+^ cells in pneumonia at 2 dpi. **a** Effect of vagotomy on *Chrna7* expression in BM cells. The bone marrow cells were collected from sham and vagotomized pneumonia groups at 2 dpi. RNA was extracted and subjected to RT-PCR. **b** Immunofluorescence images of α7nAChR and VE-cadherin expression in the bone marrow cells. Arrows indicate α7nAChR^+^VE-cadherin^+^ cells. **c** Flow cytometry analysis of VE-cadherin^+^ cells in the bone marrow from sham and vagotomized pneumonia mice at 2 dpi. **d** Immunofluorescence analysis of the coexpression of α7nAChR and VE-cadherin in the bone marrow from sham and vagotomized pneumonia mice at 2 dpi. **e** Immunofluorescence analysis of the coexpression of α7nAChR and VE-cadherin in the bone marrow from saline- and nicotine-treated pneumonia mice at 2 dpi

*Chrna7* expression in the bone marrow cells from vagotomized *E. coli* pneumonia mice compared to sham *E. coli* pneumonia mice at 2 dpi was measured by RT-PCR analysis. Vagotomized *E. coli* pneumonia mice had a lower level of *Chrna7* expression in the bone marrow cells (Fig. 4a). Immunofluorescence of the bone marrow was performed to locate the expression of α7nAChR and VE-cadherin. α7nAChR was expressed in the bone marrow vessels. More importantly, α7nAChR and VE-cadherin were coexpressed in normal bone marrow cells (Fig. 4b). Using flow cytometry analysis, we found that VE-cadherin^+^ cells among the bone marrow cells from vagotomized *E. coli* pneumonia mice were reduced compared to those in sham *E. coli* pneumonia mice (Fig. 4c). Using immunofluorescence analysis, we also found that expression of α7nAChR^+^VE-cadherin^+^ in the bone marrow cells from vagotomized *E. coli* pneumonia mice was decreased compared to that in sham *E. coli* pneumonia mice at 2 dpi (Fig. 4d). α7nAChR^+^VE-cadherin^+^ bone marrow cells from nicotine-treated *E. coli* pneumonia mice were increased compared to those in saline-treated *E. coli* pneumonia mice at 2 dpi (Fig. 4e). These findings suggest that activation of α7nAChR regulates α7nAChR^+^VE-cadherin^+^ in the bone marrow cells in *E. coli* pneumonia.

### Activation of α7nAChR regulates α7nAChR^+^p-Akt1^+^ bone marrow cells in *E. coli* pneumonia

**Fig. 5:**
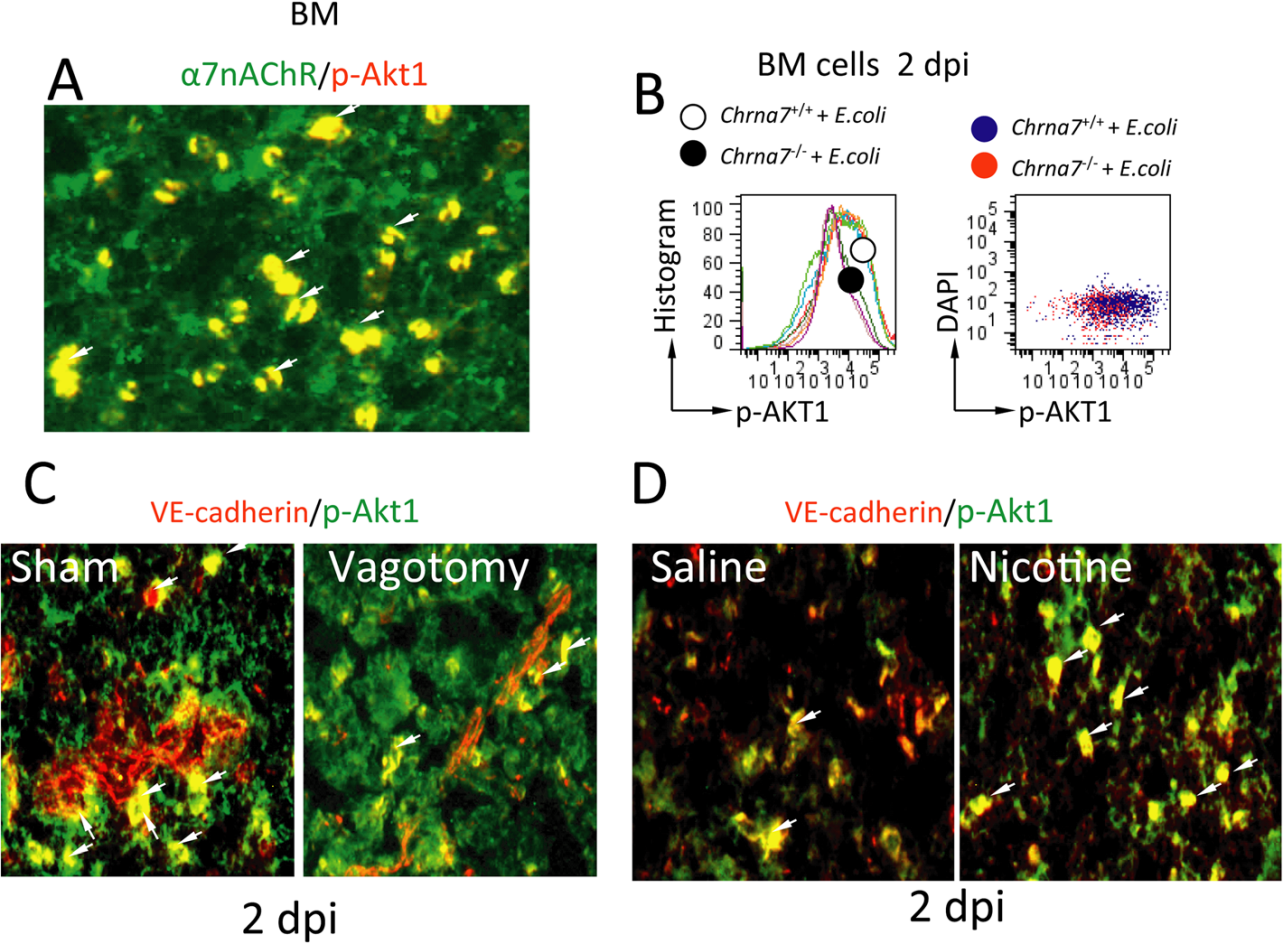
Effects of vagotomy or α7nAChR knockout on p-Akt1^+^VE-cadherin^+^ cells in pneumonia at 2 dpi. **a** Immunofluorescent images of α7nAChR and p-Akt1 expression in the bone marrow cells. **b** Flow cytometry analysis of p-Akt1^+^ cells in the bone marrow from wild-type and α7nAChR knockout mice with pneumonia at 2 dpi. **c** Immunofluorescence analysis of the coexpression of p-Akt1 and VE-cadherin in the bone marrow from sham and vagotomized pneumonia mice at 2 dpi. **d** Immunofluorescence analysis of the coexpression of p-Akt1 and VE-cadherin in the bone marrow from saline- and nicotine-treated pneumonia mice at 2 dpi

In the normal bone marrow cells, α7nAChR and p-Akt1 were coexpressed (Fig. 5a). p-Akt1^+^ cells in the bone marrow from *Chrna7*^**−/−**^ *E. coli* pneumonia were decreased compared to those in *Chrna7*^**+/+**^ sham *E. coli* pneumonia at 2 dpi (Fig. 5b). By immunofluorescent analysis, we found that p-Akt1 and VE-cadherin were coexpressed in the bone marrow. p-Akt1^+^VE-cadherin^+^ expression in the bone marrow cells from vagotomized *E. coli* pneumonia mice was decreased compared to that in sham *E. coli* pneumonia mice at 2 dpi (Fig. 5c). p-Akt1^+^VE-cadherin^+^ expression in the bone marrow cells from nicotine-treated *E. coli* pneumonia mice was increased compared to that in saline-treated *E. coli* pneumonia mice at 2 dpi (Fig. 5d).

### Deletion of *Akt1* reduces Sca1^+^ cells in the bone marrow and BAL in *E. coli* pneumonia

**Fig. 6:**
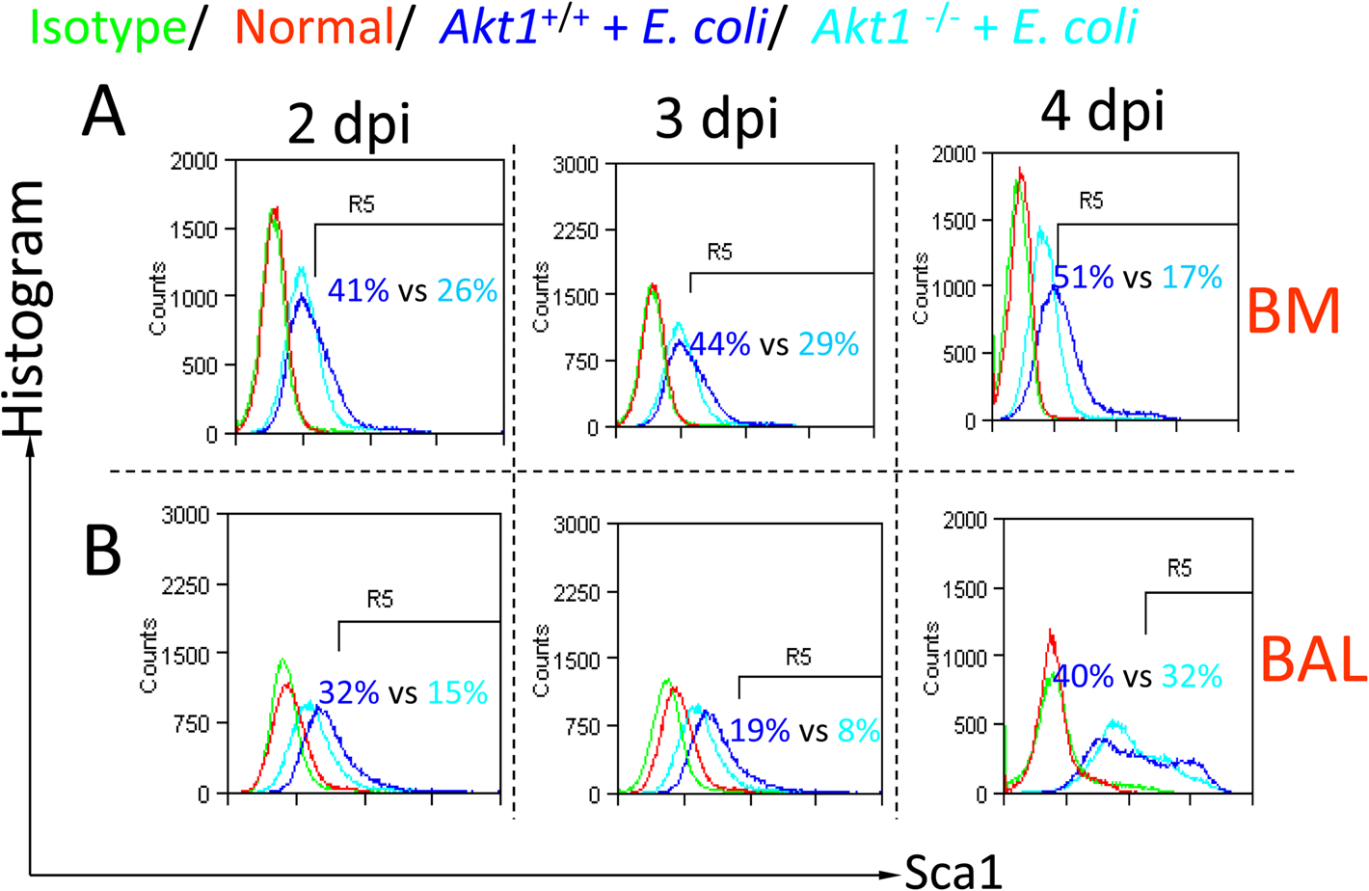
Knockout of Akt1 affects Sca1^+^ cells in the bone marrow and BAL in pneumonia. **a** Effect of Akt1 knockout on Sca1^+^ cells in the bone marrow from pneumonia mice at 2, 3, and 4 dpi. BM cells were isolated from wild-type and Akt1 knockout pneumonia mice at 2, 3, and 4 dpi for flow cytometry analysis. Normal cells were used as controls. **b** Effect of Akt1 knockout on Sca1^+^ cells in BAL from pneumonia mice at 2, 3, and 4 dpi. BAL cells were isolated from wild-type and Akt1 knockout pneumonia mice at 2, 3, and 4 dpi for flow cytometry. Normal cells were used as controls

We isolated the bone marrow and BAL cells from *Akt1* ^+/+^ and *Akt1*^−/−^ *E. coli* pneumonia mice at 2, 3, and 4 dpi to analyze Sca1-expressing cells by flow cytometry. Normal cells were used as controls. We found that the Sca1^+^ cell population was markedly reduced in the bone marrow and BAL cells from *Akt1* ^−/−^ *E. coli* pneumonia mice compared to *Akt1* ^−/−^ *E. coli* pneumonia mice at 2, 3, and 4 dpi (Fig. 6a, b). These findings support the notion that Akt1 is an important regulator of Sca1 expression in the bone marrow and BAL.

### Bone marrow and lung α7nAChR^+^Sca1^+^VE-cadherin^+^ cells express FLK-1, and vagotomy reduces lung α7nAChR^+^Sca1^+^VE-cadherin^+^ and α7nAChR^+^Sca1^+^VE-cadherin^+^p-Akt1^+^ cells

**Fig. 7:**
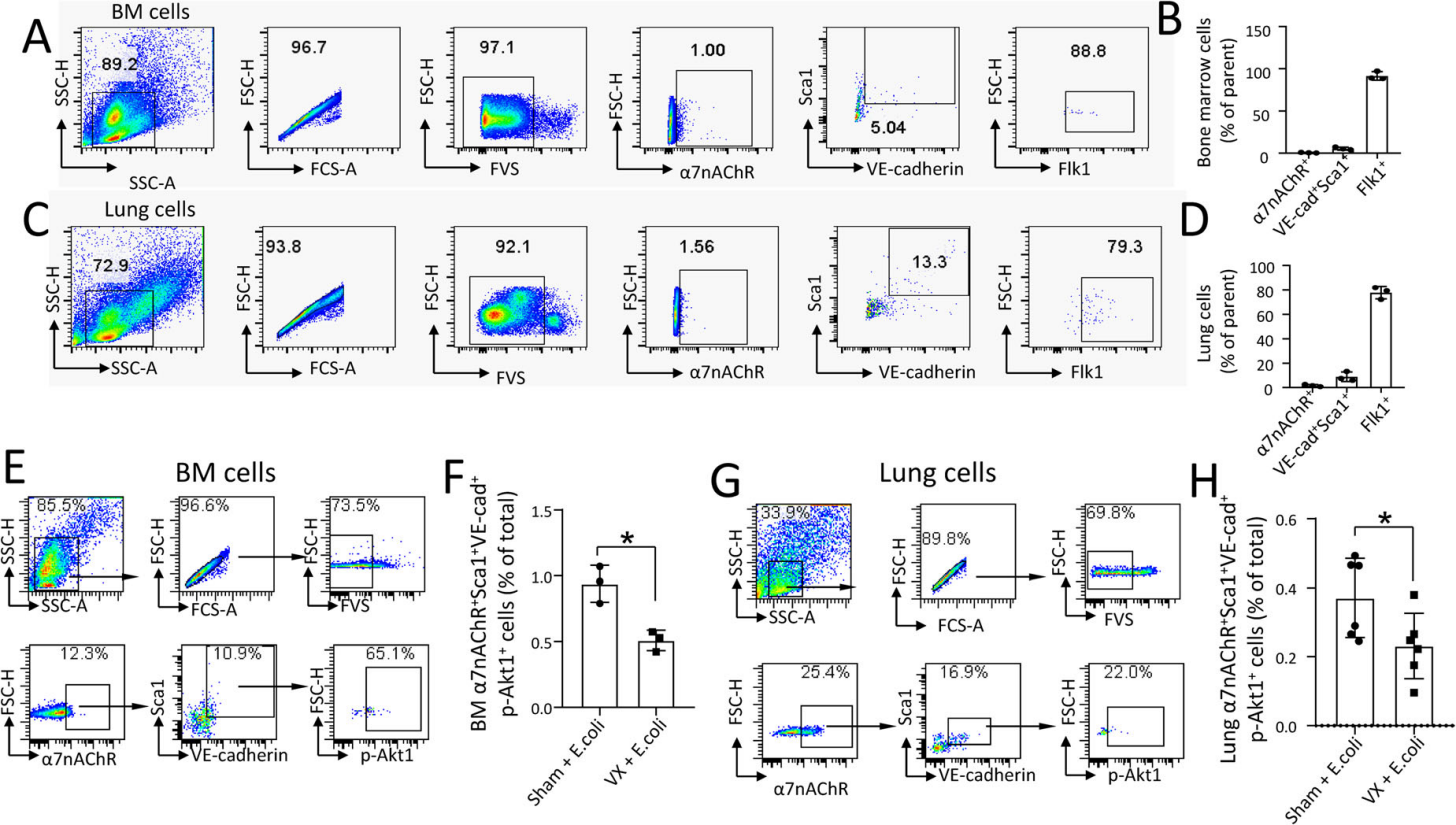
Flk1 expression in the α7nAChR^+^Sca1^+^VE-cadherin^+^ cell population and the effect of vagotomy on α7nAChR^+^Sca1^+^VE-cadherin^+^p-Akt1^+^ cells in BM and lungs from sham and vagotomized mice. **a** Flow cytometric analysis of α7nAChR^+^Sca1^+^VE-cadherin^+^Flk1^+^ cells in normal bone marrow cells. **b** The cell percentage analyzed from Fig. 7a. **c** Flow cytometric analysis of α7nAChR^+^Sca1^+^VE-cadherin^+^Flk1^+^ cells in normal lung cells. **d** The cell percentage analyzed from Fig. 7c. **e**, **f** Effect of vagotomy on α7nAChR^+^Sca1^+^VE-cadherin^+^p-Akt1^+^ cells in BM cells. *N* = 3 in each group. Student’s *t* test, *P < 0.05. **g**, **h** Effect of vagotomy on α7nAChR^+^Sca1^+^VE-cadherin^+^p-Akt1^+^ cells in lung cells. *N* = 6 in each group. Student’s *t* test, *P < 0.05

Since Sca1^+^Flk-1^+^ cells are considered endothelial progenitor cells (EPCs), we analyzed whether the bone marrow and lung α7nAChR^+^Sca1^+^VE-cadherin^+^ cells express Flk-1 (VEGFR2). We collected the bone marrow and lung cells to detect α7nAChR^+^Sca1^+^VE-cadherin^+^Flk1^+^ cells in normal mice. We found that 91.3 ± 4.9% of BM and 77.8 ± 4.9% of lung α7nAChR^+^Sca1^+^VE-cadherin^+^ cells were Flk1^+^ (Fig. 7a–d). These findings indicate that most α7nAChR^+^Sca1^+^VE-cadherin^+^ cells could be EPCs, which possess lung injury repair potential. Lung α7nAChR^+^Sca1^+^VE-cadherin^+^ cells were reduced in vagotomized *E. coli* pneumonia mice compared to sham *E. coli* pneumonia mice at 3 dpi (Fig. 7c). To compare the p-Akt1 levels in α7nAChR^+^Sca1^+^VE-cadherin^+^ cells between sham and vagotomized *E. coli* pneumonia mice, we collected BM and lung cells for flow cytometry. We found that BM α7nAChR^+^Sca1^+^VE-cadherin^+^p-Akt1^+^ cells were reduced in vagotomized *E. coli* pneumonia mice compared to sham *E. coli* pneumonia mice at 3 dpi (Fig. 7e–f). The lung α7nAChR^+^Sca1^+^VE-cadherin^+^p-Akt1^+^ cells were also reduced in vagotomized *E. coli* pneumonia mice compared to sham *E. coli* pneumonia mice at 3 dpi (Fig. 7g–h). These findings indicate that vagal circuits regulate Akt1 phosphorylation in α7nAChR^+^Sca1^+^VE-cadherin^+^ cells.

### Activation of α7nAChR in EPCs reduces ELW in *E. coli* pneumonia mice

**Fig. 8:**
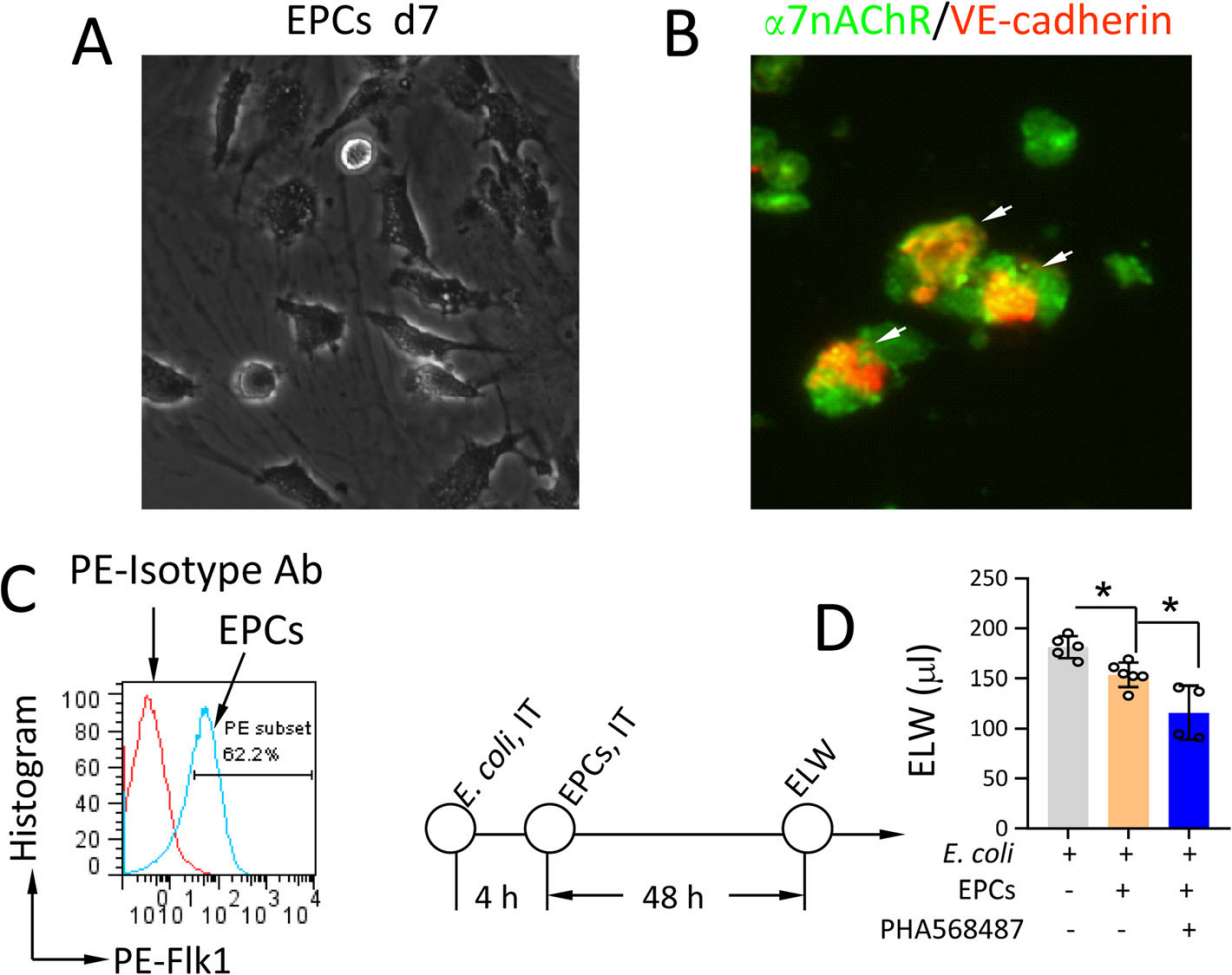
Effects of activation of α7nAChR in cultured EPCs on pulmonary edema in *E. coli* pneumonia mice. **a** Morphology of cultured EPCs at d7 and d14. **b** Immunofluorescent detection of α7nAChR and VE-cadherin expression in the culture EPCs. Arrows indicate α7nAChR^+^VE-cadherin^+^ cells. **c** Flow cytometric analysis of Flk1 expression in cultured EPCs. **d** Effect of activation of α7nAChR in EPCs on lung water in *E. coli* pneumonia mice. One-way ANOVA, *P < 0.05, *N* = 4–6 in each group

As documented by light microscopy, the attached cells adopted a spindle-shaped or cord-like morphology after 7 days in culture (Fig. 8a). About half of the cells were α7nAChR^+^VE-cadherin^+^ positive in immunofluorescent staining (Fig. 8b). Confirmed by flow cytometry, these cells were 62.2% Flk1-positive (Fig. 8b). Treatment with EPCs (intratracheal (IT) delivery, 2.5 × 10^5^/mouse) 4 h after *E. coli* infection (induced by IT *E. coli*, 2.5 × 10^5^ CFU) reduced ELW compared to PBS-treated *E. coli* pneumonia mice at 48 h. The ELW was further reduced by treatment with EPCs (2.5 × 10^5^) combining with α7nAChR agonist-PHA568487 (0.4 mg/kg, ip, q6h) compared to treatment with EPCs only. These findings suggest that activation of α7nAChR in EPCs is protective for *E. coli* pneumonia by reducing lung vascular permeability.

## Discussion

In this study, for the first time, we determined that vagal circuit α7nAChR signaling could regulate α7nAChR^+^Sca1^+^ cells, especially α7nAChR^+^Sca1^+^VE-cadherin^+^ cells, in the bone marrow and lung during pneumonia. The bone marrow and lung α7nAChR^+^Sca1^+^VE-cadherin^+^ cells are Flk1^+^ EPCs that could be regulated by phosphorylation of Akt1.

The distal airways of the lung are innervated by the vagus nerve. Vagal α7nAChR signaling plays a key role in regulating lung infection and inflammation [2, 6, 35]. Vagal α7nAChR signaling could also regulate the proliferation and transdifferentiation of lung stem cells (LSCs) and promote lung injury repair [9]. Vagotomy or α7nAChR deficiency reduced lung Ki67^+^ LSC expansion and hampered the resolution of LPS-induced lung injury. Vagotomy or α7nAChR deficiency decreased lung fibroblast growth factor 10 expression and the number of type II alveolar epithelial cells. In addition, our previous studies confirmed that vagotomy augmented splenic egress and lung recruitment of α7nAChR^+^CD11b^+^cells, which was another mechanism that contributed to worsened lung inflammatory responses. Monocytes and neutrophils recruited to the lung were also contributed to the pathogenesis of LPS or *E. coli* lung injury in α7nAChR knockout mice with [2, 6, 35]. Thus, it is hard to exclude the effect of vagotomy caused by other system changes besides lung responses in this study. We would further investigate the point in future work. However, the findings in this study are consistent with our previous investigations, which demonstrate that vagal α7nAChR signaling regulates lung inflammation and injury repair.

We found that Sca1^+^ or α7nAChR^+^Sca1^+^ cells were increased in the bone marrow, blood, and BAL, and these changes were attenuated by vagotomy or Akt1 deficiency. Thus, so far, we could not answer the question of whether the bone marrow is the source of lung Sca1^+^ or α7nAChR^+^Sca1^+^ cells during lung injury repair; however, the findings that transplantation of α7nAChR^+^Sca1^+^ cell-enriched BM-MNCs to recipient pneumonia lungs improved lung injury and increased engraftment indicate that α7nAChR^+^Sca1^+^ cells might migrate into the lung for reparative processes. To prove this, a bone marrow reconstitution experiment might be needed in future studies. Determining the chemotactic receptor of BM α7nAChR^+^Sca1^+^ cells and blockade of chemotaxis of these cells during the repair process are possible approaches. For instance, CXCR2 is critical for both EPC recruitment and the angiogenic response in a model of allergic inflammation of the airways [38].

In the bone marrow, α7nAChR was expressed in the large vessels (Fig. 4b), but these vessels expressed less VE-cadherin. In some VE-cadherin-expressing small vessels, α7nAChR expression was negative (Fig. 4e). Coexpression of α7nAChR and VE-cadherin was found in the periphery of blood vessels or capillaries in the bone marrow. The distribution of α7nAChR^+^ VE-cadherin^+^ cells might facilitate the egress of these cells during lung injury repair.

It has been reported that EPC transplantation could improve LPS-induced acute lung injury [27, 39–41]. In 23 patients with pneumonia, the proportion of EPCs in peripheral blood MNCs was increased. Patients with low EPC numbers tended to have persistent fibrotic changes in their lungs even after their recovery from pneumonia [42]. In our study, we identified α7nAChR^+^Sca1^+^VE-cadherin^+^Flk1^+^ EPCs in the bone marrow and lung during pneumonia. These findings have laid a basis for us to understand how the vagus nerve regulates EPCs via α7nAChR during lung infection and injury.

Our results are also supported by the observation that nicotine treatment could enhance the number and functional activity of EPCs [17, 43, 44]. Mechanistically, nicotine could dose-dependently prevent the onset of EPC senescence via the PI3K/Akt pathway [21]. In our study, we determined that vagal α7nAChR signaling regulates Akt1 phosphorylation in the bone marrow cells. Knockout of Akt1 could reduce Sca1^+^ cells in the bone marrow, blood, and BAL in pneumonia mice. We have provided direct evidence that Akt1 is a key regulator of EPC repair during pneumonia.

Since *E. coli* pneumonia is not a suitable model to test angiogenesis, we have to use excess lung water as a surrogate of lung vascular permeability to examine the functionality of EPCs. We developed EPCs and administrated these cells to the *E. coli* pneumonia mice. We found that treatment with EPCs reduced ELW compared to that in PBS-treated *E. coli* pneumonia. In combination with α7nAChR agonist-PHA568487, EPCs could further reduce ELW in *E. coli* pneumonia. Previous studies have also showed that BMPC transplantation or mobilization of BMPCs can induce endothelial barrier protection by interfering with signaling pathways in endothelial cells mediating the increased permeability in response to proinflammatory mediators [25, 36]. In vivo EPC administration in sepsis increased plasma IL-10, suppressed lung vascular leakage, attenuated liver and kidney injury, and augmented miR-126 and -125b expression, which regulates endothelial cell function and/or inflammation [28]. Transplantation of EPCs provides a modest benefit for treatment of the ischemic diseases such as limb ischemia [45]. EPCs may be capable of directly engrafting and regenerating the endothelium, the most important effects of EPCs seem to be dependent on paracrine effects [46, 47].

## Conclusion

We identified the bone marrow and lung α7nAChR^+^Sca1^+^VE-cadherin^+^ Flk1^+^ EPCs. During pneumonia, these cells could be regulated by vagal α7nAChR signaling via phosphorylation of Akt1.

## Data Availability

All data generated or analyzed during this study are included in this published article.

## Abbreviations

ARDS: Acute respiratory distress syndrome
α7nAChR: Alpha7 nicotinic acetylcholine receptor
Sca1: Stem cell antigen 1
BM: Bone marrow
BM-MNCs: Bone marrow mononuclear cells
MSCs: Mesenchymal stem cells
EPCs: Endothelial progenitor cells
LPS: Lipopolysaccharide
RT-PCR: Real-time polymerase chain reaction
PE: Phycoerythrin
FITC: Fluorescein isothiocyanate
Vx: Vagotomy
PBS: Phosphate buffer saline
